# Effect of a High Linoleic Acid Diet on Pregnant Women and Their Offspring

**DOI:** 10.3390/nu16173019

**Published:** 2024-09-06

**Authors:** Deepti Nayyar, Joanne M. Said, Helen McCarthy, Deanne H. Hryciw, Lannie O’Keefe, Andrew J. McAinch

**Affiliations:** 1 Institute for Health and Sport, Victoria University, P.O. Box 14428, Melbourne, VIC 8001, Australia; deepti.nayyar@live.vu.edu.au (D.N.); helen.mccarthy1@vu.edu.au (H.M.); d.hryciw@griffith.edu.au (D.H.H.); lannie.okeefe@vu.edu.au (L.O.); 2 Department of Maternal Fetal Medicine, Joan Kirner Women’s & Children’s Sunshine Hospital, Western Health, St Albans, VIC 3021, Australia; jsaid@unimelb.edu.au; 3 Department of Obstetrics, Gynaecology and Newborn Health, The University of Melbourne, Parkville, VIC 3052, Australia; 4 School of Environment and Science, Griffith University, Nathan, QLD 4111, Australia; 5 Griffith Institute for Drug Discovery, Griffith University, Nathan, QLD 4111, Australia; 6 Australian Institute for Musculoskeletal Science (AIMSS), Victoria University, P.O. Box 14428, Melbourne, VIC 8001, Australia

**Keywords:** nutrition, linoleic acid, alpha linoleic acid, birth weight, inflammatory markers, placenta

## Abstract

Nutritional intake during pregnancy can affect gestational length, fetal development, and impact postnatal growth and health in offspring. Perturbations in maternal nutrition with either an excess or deficiency in nutrients during pregnancy may have harmful effects on the offspring’s development and increase the risk of developing chronic diseases later in life. In pregnancy, nutrients transfer from the mother to the fetus via the placenta. Essential fatty acids, linoleic acid (LA) and alpha linoleic acid (ALA), can only be obtained in the diet. In Western countries, the ratio of LA and ALA in the diet has increased dramatically in recent decades. Some animal and human studies have found a correlation between maternal intake of LA and birth weight; however, the association varies. In contrast, some human studies have demonstrated inconclusive findings regarding the correlation between cord blood levels of LA and birth outcomes. In addition, high dietary LA intake in animal studies in pregnancy increased the production of inflammatory markers such as prostaglandins, leukotrienes, cytokines, and tumour necrosis factor-alpha. This review aims to highlight the effect of high dietary LA intake during pregnancy on birth outcomes, obesity, maternal inflammatory markers, and the transfer of fatty acids across the placenta.

## 1. Introduction

Nutritional intake during pregnancy can affect gestational length, fetal length and weight, and thus, health in the developing offspring [1,2]. Perturbations in maternal nutrition during pregnancy with either an excess or deficiency may have harmful effects on the offspring’s health [3,4] and increase the risk of developing chronic diseases later in life [5]. During pregnancy, there is an increased demand for macronutrients and micronutrients, which are transported from the mother to her fetus via the placenta [6]. A balanced diet is required to maintain the needs of the growing fetus, and healthy placental development, as well as the increased requirements associated with the maternal adaptations to pregnancy [7].

The principal metabolic nutrients required by the fetus are glucose and amino acids [8]. Glucose is the principal energy substrate for basal metabolism and protein synthesis and contributes to energy storage in glycogen and fat [8]. Amino acids provide the building blocks for protein synthesis and growth, especially when glucose is deficient [8]. Inappropriate amounts of macronutrients and micronutrients such as carbohydrates, proteins, fats, folate, thiamine, vitamin C, niacin, iron, and vitamin B12, which are also critical, may predispose the offspring to chronic conditions later in life such as obesity, type 2 diabetes, cardiovascular disease, and neurodevelopmental delays [9]. 

In addition to these micronutrients, fatty acids (FAs), in particular linoleic acid (LA) and alpha-linolenic acid (ALA), play crucial roles in cell membrane formation and neurodevelopment in fetus [10]. LA is a precursor to arachidonic acid (AA), which is integral to brain and retinal development [11]. Similarly, ALA serves as a precursor to eicosapentaenoic acid (EPA) and docosahexaenoic acid (DHA), essential for the development of the fetal brain and retina [12]. However, excessive LA relative to ALA in the diet can disrupt the balance of essential fatty acids in the fetal brain, potentially impairing cognitive and neurological development [13]. Additionally, high LA maternal dietary levels can promote inflammation, impact cardiovascular health, and affect fat metabolism and growth patterns, influencing birth weight and the risk of obesity and metabolic disorders in offspring [14]. This review will provide a brief discussion of the importance of specific micronutrients and macronutrients before progressing to a detailed discussion of the impact of LA and ALA in fetal development and long-term health.

## 2. Micronutrients

Micronutrients, vitamins and minerals, are essential nutrients that are required for normal body functioning [15]. Before and during pregnancy the requirements of specific micronutrients are increased [16]. Maternal nutritional intake in the first trimester may be more important to the development and differentiation of various organs, whereas diet later in pregnancy may be important for overall fetal growth as well as for brain development [17]. During the first trimester, the requirement of folate increases substantially due to its role in nucleic acid synthesis [18]. Adequate folate is required for the prevention of neural tube defects, and supplementation prior to conception and in the first trimester of pregnancy decreases the risk of neural tube defects [19]. Along with folate, adequate vitamin B12 is important during pregnancy for deoxyribonucleic acid synthesis (DNA) and various neurological functions such as brain development and neural myelination [20,21]. Further, infants born to mothers with low serum vitamin B12 in the second trimester of pregnancy are more likely to have lower birth weight (<10th percentile—World Health Organization) [22]. 

Thiamine (vitamin B1) plays a vital role in the supply of energy to the tissues, and in the metabolism of carbohydrates, proteins, and fats [4]. During pregnancy, the requirement for thiamine increases by 30% [23]. To cover increased energy utilization and growth during pregnancy, an additional 3 mg/day of niacin (vitamin B3) is required [24]. Epigenetic regulatory enzymes such as histone demethylases, crucial for embryonic development, have been identified to depend on vitamin C as a cofactor, underscoring the significance of maternal dietary vitamin C intake [25]. 

Iron is an important component of many proteins such as hemoglobin, myoglobin, and enzymes [7]. Iron requirements progressively increase in pregnancy and peaks during the third trimester of pregnancy (3–7.5 mg/day) [16,26]. There is a significant increase in iron requirement from 5–8 mg/day in non-pregnant women to 22–23 mg/day in pregnant women [7]. A deficiency of iron during pregnancy may lower the delivery of oxygen to the maternal tissues, resulting in pallor, fatigue, fainting, and breathlessness [27]. Moreover, iron deficiency during pregnancy may negatively impact perinatal outcomes such as premature labour, intrauterine growth restriction, low birth weight, birth asphyxia, and infant anemia [27]. 

## 3. Macronutrients

Macronutrients, namely carbohydrates, proteins and fats, are those nutrients that are required in large amounts in the diet to enable the growth and repair of tissues and maintain bodily functions [28]. In Australia and New Zealand, the recommended daily intake for adults is 45–65% of one’s energy from carbohydrates, 15–25% from proteins, and 20–35% from fats [29]. Energy expenditure increases during pregnancy to account for the growing fetus. However, it does not increase immediately as energy requirements are the same as non-pregnant women in the first trimester, and then increase by an estimated 1400 kJ (340 kcal)/day in the second trimester and 1900 kJ (452 kcal)/day in the third trimester [30]. Among the macronutrients, protein requirements increase, especially later in the pregnancy. To meet these demands, the Estimated Average Requirement (EAR) of protein during the second and third trimester of pregnancy is 1.1 g/kg/body weight/day compared to 0.8 g/kg/body weight/day during first trimester of pregnancy [31]. Excessive or insufficient maternal dietary protein intake can cause heritable changes in gene expression during fetal development by affecting DNA methylation [32]. Low maternal dietary protein intake during pregnancy can also result in intrauterine growth restriction (IUGR) and reduced postnatal growth in humans [33]. Furthermore, individuals who experienced IUGR due to maternal dietary protein restriction may be predisposed to a higher risk of metabolic disorders such as type 2 diabetes and cardiovascular disease in adulthood [34].

## 4. Fatty Acids

From conception to the first two years of life, fats serve as the primary energy source in an infant’s diet, playing a critical role in the growth, development, and overall long-term health [35]. Fats can be categorized into essential fatty acids (EFAs) and non-essential fatty acids. FA are an important component of cell membranes and organelles and are a precursor of many hormones and metabolic regulators, which are necessary for a healthy pregnancy [36,37]. EFAs are derived from the diet and cannot be synthesized by the body due to the absence of delta 12 (Δ12) and delta 15 (Δ15) desaturase enzymes [38]. LA and ALA are EFAs important for human health [39]. The availability of these EFAs to the fetus depends upon the maternal transfer across the placental membrane [40]. EFAs play a vital role in the body as a structural and functional component of a cell membrane, modulating cell signalling, gene expression, and inflammation [41]. EFAs act as the precursors for eicosanoid and docosanoid production (prostaglandins, thromboxane, and leukotrienes), which have important bio regulatory functions, such as thrombocyte aggregation, inflammatory responses, vasoconstriction, and vasodilatation [42]. 

## 5. Linoleic Acid

LA and ALA are eighteen-carbon polyunsaturated fatty acids (PUFA), which can be desaturated and elongated into a series of longer chain unsaturated fatty acids through enzymatic action [43]. High dietary intake of LA may lead to overproduction of AA, which in turn triggers the production of inflammatory compounds such as prostaglandin E2, leukotriene B4 and thromboxane [44]. LA and ALA compete for the enzymes responsible for fatty acid chain elongation and desaturation resulting in decreased production of EPA and DHA [45,46]. LA is predominantly obtained through the consumption of vegetable oils (sunflower, safflower, corn, soya bean, peanut oil, and palm oil), chicken, eggs, meats, processed foods, and nuts [47]. ALA is mainly obtained from plant oils such as canola and soyabean oils; seeds and nuts such as flaxseed, chia, and walnut; and some green leafy vegetables such as kale and spinach [48,49]. 

The World Health Organization recommends that LA at a level of 2% of energy intake fulfils EFA needs [50]. However, 1–2% of dietary energy from LA is enough to prevent deficiency symptoms such as growth retardation, infertility, skin desquamation [51]. Over the last few decades, there has been a substantial increase in the availability of LA in the diet throughout the Westernized world. Before 1930, the amount of LA in the diet in the United States (US) was between 2 and 4 g/day (1–2% of daily energy intake), which has now increased to 19 g/day (7% of daily energy intake) [46]. In Australia, the intake of LA in the diet has increased from 2.2% to 6% of total available energy between 1991 and 2009 [52]. This may be due to an increase in consumption of plant-based vegetable oils [53], processed foods with increased shelf life [54], and ready-to-eat foods, as these foods are easy to access and cheaper to purchase [47]. Usage of soya bean oil, a main source of LA, has increased ~1000-fold per capita over the past century in American diets [46]. In Australia, from 1961 to 2009, there has been a significant increase in the availability of foods high in LA such as cottonseed oil, peanut oil, palm oil, rapeseed oil, soya oil, sunflower, wheat, poultry, eggs, pork, lamb, milk, and beef [52]. 

**Figure 1:**
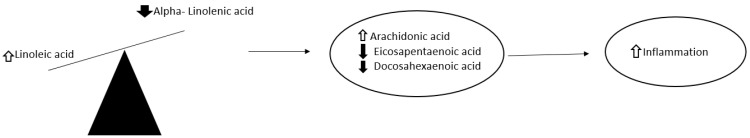
Effect of dietary increase in the ratio of linoleic acid and alpha-linoleic acid on inflammation. Arrow pointing up means an increased amount, while the arrow pointing down means a decreased amount.

As LA and ALA are metabolized by the same enzymes, an increased consumption of LA decreases the conversion of ALA to EPA and DHA due to the downregulation of fatty acid desaturase 1 (FADS1), affecting their availability for cells [55]. The optimal ratio of LA to ALA in the diet is 1:1 or 2:1 [56], whereas the current excess LA in the diet has increased the ratio of LA to ALA from 4:1 to 20:1 [57]. The interference in the production of EPA and DHA from ALA, may therefore be a contributing factor for health implications of excess LA intake, as ALA has beneficial effects on cardiovascular [58] and inflammatory diseases [59] (Figure 1). 

## 6. Effect of LA on Inflammation

LA metabolism results in the production of AA, and downstream pro-inflammatory eicosanoids such as prostaglandins, leukotrienes, and thromboxane [60]. Increased levels of pro-inflammatory eicosanoids enhance the level of biomarkers of inflammation such as interleukin-6, tumour necrosis factor alpha (TNF-α), and C-reactive proteins that are associated with increased incidence of chronic diseases such as cardiovascular diseases [60]. In contrast, metabolites of ALA have anti-inflammatory effects [56]. LA is metabolized by 5–lipoxygenase and causes inflammation by enhancing production of pro-inflammatory lipoxins and through synthesis of AA [45,61]. AA is metabolized to various lipid mediators such as eicosanoids and prostaglandins, also known as oxylipins and hydroxyoctadecadienoic acids (HODEs), oxo-HODEs and epoxy-HODEs [62]. AA is converted into various inflammatory metabolites, such as cytochrome P450, cyclooxygenase, and lipoxygenase pathways [63]. 

LA and ALA may exert their effects on inflammation through intricate epigenetic mechanisms that modulate gene expression [64]. Eicosanoids produced through the conversion of LA to AA can affect gene expression by interacting with nuclear receptors and transcription factors, leading to an inflammatory response [65]. Conversely, ALA is metabolized into anti-inflammatory eicosanoids, including resolvins and protectins, to counteract inflammation and promote resolution [65]. ALA’s anti-inflammatory effects are mediated through its influence on histone acetylation and DNA methylation patterns, downregulating pro-inflammatory cytokines and upregulating anti-inflammatory genes [66].

As determined via meta-analysis, no relationship was found between LA and increased inflammation in healthy human beings [67]. In contrast however, a positive relationship between LA and increased inflammation in individuals with cardiovascular diseases was observed in another meta-analysis [61]. As the availability of LA in Western cultures has led to increased consumption [68], it is therefore important to assess the effect of high dietary LA on maternal inflammatory markers during pregnancy. 

**Figure 2:**
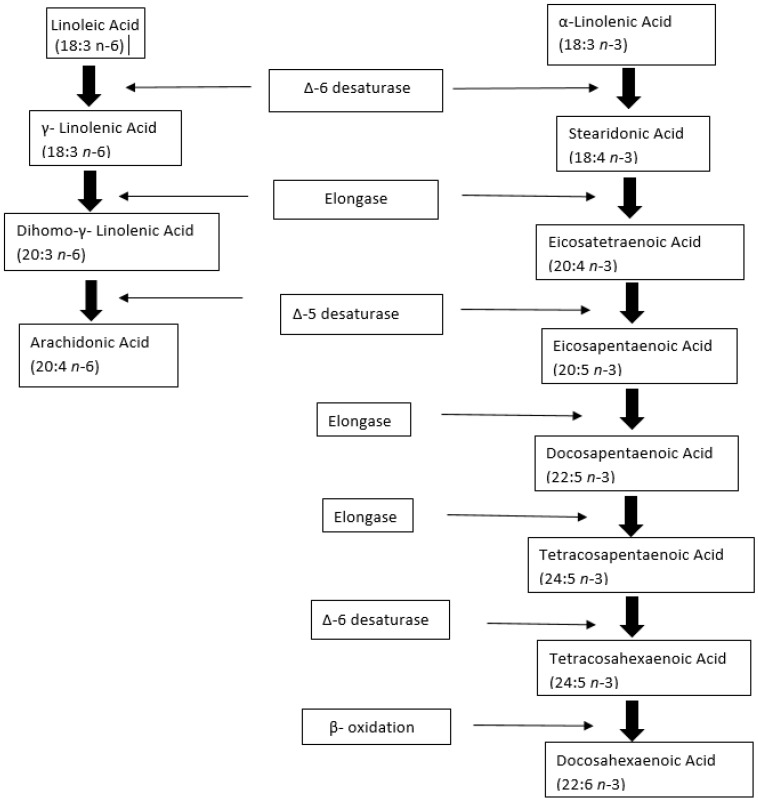
Metabolism of linoleic acid (LA) and α-linolenic acid (ALA) to a series of longer-chain unsaturated fatty acids, through enzymatic action. ∆-6 desaturase, Elongase, and ∆-5 desaturase are the common enzymes in the metabolism of LA and ALA. Arachidonic acid is produced from LA and Docosahexaenoic acid from ALA. Adapted from [73].

An investigation in animals suggests that the weight gaining property of LA is due to synthesis of AA from LA which occurs through the prostacyclin pathways [69] (Figure 2). AA is the precursor for 2-Arachidonoylglycerol (2-AG) and N-arachidonoylethanolamine (anandamide or AEA) [11]. Weight is increased in pups of rodent dams fed with a 7% LA diet compared to 7% ALA diet at 3 weeks of age, showing the importance of LA in the maternal diet for offspring adiposity [70]. In agreement with this, rats fed on a high LA diet (18% of total energy) and low ALA (0.6% of total energy) without any difference in saturated fats increased weight in each successive generation [69]. However, the amount of LA in the animal diet was 18% of total energy intake [69] compared to human dietary consumption of LA, being ~7% of daily energy intake in a Western diet [46]. It is also found that the impact of a high LA:ALA diet (9:1) on fetal weight in Wistar rats was different for males and females, as female fetuses were heavier in the low LA:ALA diet (1:1.5) group irrespective of dietary fat content [71]. However, we found that no increase in fetal weight was observed in rats when fed a LA (6.21% of energy) diet for 10 weeks before mating and 20 days during gestation [72]. 

## 7. Effect of LA on Fetal Development and Leptin

There is a strong association between LA and leptin in vitro and in animals [72,73]. Fetal development has also been found to be influenced by the adipokine leptin, which is responsible for regulation of maternal metabolic conditions and fetal growth in animals [74]. While LA (1–200 µM) has no effect on basal leptin production in isolation, in the presence of insulin, leptin secretion was significantly decreased in isolated rat adipocytes [75]. Furthermore, in the presence of insulin and at the highest concentration (200 µM), LA significantly reduced adiponectin secretion in rat adipocytes [75], thus suggesting that LA may interfere in insulin signalling pathways involved in the production of leptin and adiponectin [73,75]. In support of this in an animal study, a high maternal dietary intake of LA (6.21% of energy) decreased plasma leptin concentration in rodents via down regulating leptin messenger ribonucleic acid (mRNA) expression in adipose tissues [72]. In contrast, changing dietary LA intake from 1% to 8% of total energy increased leptin levels and decreased adiponectin levels in pregnant mice [76]. Furthermore, high maternal dietary LA (7% sunflower oil) in suckling rodent pups increased serum leptin levels at week 1 compared with pups of 3 weeks of age; however, suckling pups fed on a high ALA diet (7% linseed oil) showed decreased serum leptin levels from week 1 to 3 [70]. Further work therefore needs to be undertaken, particularly in humans, on the role of maternal dietary intake of LA on leptin regulation.

## 8. LA and Birth Weight

In contrast to animal studies, the association between LA intake and birth weight in humans, shows an inverted U-shaped correlation [77]. In South Indian women, a low birth weight was found at both low (<4% of energy) and high intake (>8% of energy) of maternal dietary LA [77]. Furthermore, in the Amsterdam Born Children and their Development cohort, infants born to mothers with a high dietary LA intake in early pregnancy were associated with decreased birth weight [78]. Increased dietary intake of LA negatively impacted the circulating erythrocytes ALA level in mothers who delivered low birth weight babies (<2.5 kg) as compared to those delivering normal birth weight babies (>2.5 kg) [79]. An inverse relationship between maternal intake of LA and birth weight was also found in pregnant women from Korea [80]. Moreover, Danish pregnant women who consumed a diet rich in LA have increased rates of small gestational age babies (SGA) [81]. In the United Kingdom, a positive association with dietary LA intake at 34 weeks of gestation and body fat in the offspring at 4 to 6 years of age was found, whereas in a birth cohort in the US, a high dietary intake of LA showed no obvious effect on offspring adiposity measures at the age of 3 years [82]. 

In humans, an increased maternal dietary intake of both LA (10.73 ± 6.34 g per day) and ALA (1.47 ± 1.45 g per day) showed positive relationship with low birth weight babies indicating that high LA intake may inhibit the positive effects of ALA on fetal growth [80]. Furthermore, an increased ratio of LA and ALA (from 1:1.5 to 9:1) during pregnancy, has resulted in decreased placental blood flow and increased the blood viscosity resulting in inadequate fetal growth and various metabolic diseases in infants and adulthood [45,71]. 

Ethnic differences, as well as genetic and environmental factors, could all contribute to inconsistencies in observations regarding the effects of LA on birth weight [83,84]. Understanding how these factors interact and affect pregnancy outcomes is crucial. Therefore, further studies are needed to delve deeper into the relationship between a high intake of LA in the diet and its effects on pregnancy. 

## 9. LA and Birth Length

Along with birth weight, infant’s birth length is also a crucial predictor of long term health [85]. Higher LA intake by the mother at 24 weeks of pregnancy is linked to shorter femur lengths in the infant, thought to be due to the effect of LA on bone mineralization [86]. The impact of nutritional fatty acid study (INFAT-study) in mothers from the Netherlands suggested that maternal high dietary intake of LA and ALA during the third trimester of pregnancy resulted in an increase in birth length [87], whereas a study in pregnant women from Mexico showed an inverse association of maternal second trimester PUFA intake with birth length [88]. These findings suggest that there is a relationship between dietary intake of LA during pregnancy and fetal growth; therefore, it is essential to demonstrate the effect of LA intake during pregnancy on the length of babies at birth as well as their subsequent growth.

## 10. Effects of High LA on Transfer of FA through the Placenta

The life span of erythrocytes is 120 days, and their FA concentration reflects FA metabolism over a longer time period than plasma and serum FAs [89]. Where serum FA levels reflect intake over the past weeks, erythrocytes reflect dietary intake over the past months [90]. PUFAs are transferred via the placenta during pregnancy to fulfil increased fetal requirements for EFAs during pregnancy [91]. However, in vitro perfusion in third trimester human placenta following birth demonstrated that only 20% of the estimated FA requirements are satisfied by placenta transfer, and the rest come from de novo synthesis by the fetus [92]. LA is an EFA which the mother can obtain only from her diet, and the fetus receives it from the mother; therefore, measuring the amount of LA in the umbilical cord will measure the amount of FAs transferred across the placenta to the fetus [93]. As such a high level of PUFAs at birth is positively associated with FA content of maternal diet during pregnancy [94]. FA levels that are accumulated in fetal tissue lipids and placenta during pregnancy continue to circulate in the fetal tissues and blood stream, suggesting that plasma fatty acid levels in the early stages of infancy depends upon the maternal circulating plasma fatty acid levels throughout pregnancy [43]. PUFAs in the blood can be assessed in several sample types including red blood cells, white blood cells, plasma, and platelets [95]. Some studies have examined only maternal erythrocyte FA levels [96] or maternal plasma FA levels [97] during the pregnancy without considering the FA transport through the placenta [98]. 

Pregnancy has been described as a “physiologic systemic inflammatory response” with an increase in the number of leukocytes and interleukins [99]. However, maternal weight, hormone imbalance, and increased inflammatory markers can influence the FA transport across the placenta, resulting in an effect on fetal health during pregnancy [98]. This is supported by animal studies where a high LA diet (6.21% of energy) in Wistar rats increased the production of circulating pro-inflammatory leukotrienes, prostaglandins, and cytokines such as tumour necrosis factor alpha (TNF-α) and interleukin-1β (IL-1β) in the mother in pregnancy.

## 11. Placental LA Transport

**Figure 3:**
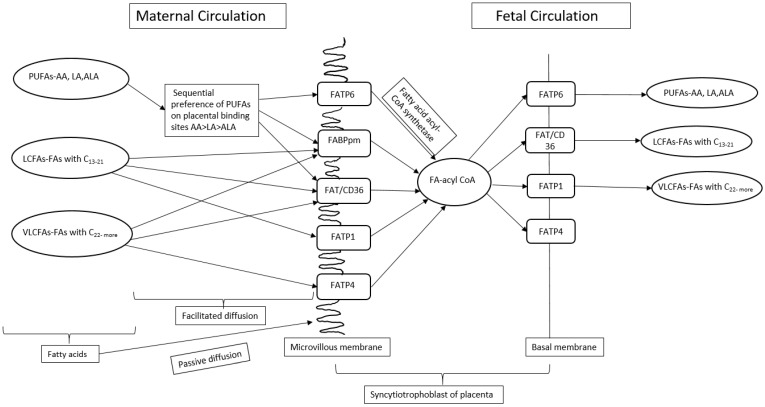
A schematic view of transport of fatty acids across placenta during pregnancy. PUFAs—Polyunsaturated fatty acids, LA—Linoleic acid, ALA—Alpha linoleic acid, AA—Arachidonic acid, LCFAs—Long chain fatty acids, VLCFAs—Very long chain fatty acids, C—carbon, FA-acyl-CoA—Fatty acid acetyl coenzyme A, FATP—Fatty acid transport proteins, FABPpm—Plasma membrane fatty acid binding proteins, FAT/CD36—Fatty acid translocase.

The transfer of FA across the placenta during pregnancy from mother to fetus requires FA carriers/transporters [100]. Lipid uptake requires the release of esterified FA within maternal lipoproteins [101]. Free FAs are more readily taken up by the placenta [102], which are transported from the mother to fetus through FA transport proteins (FATPs), fatty acid translocase (FAT), and intracellular FA binding proteins (FABPs) present in the placenta [98] (Figure 3). It has been observed that the binding of maternal FA with FATPs, present on the microvillus membrane of syncytiotrophoblast, are endocytosed and transported to the basolateral FATPs [103]. 

Subsequently, FAs enter the fetal circulation by binding with the fetal FATPs present in the fetal endothelial capillary cell membrane [103]. FA binding sites exhibit a hierarchy of preference for PUFAs, with AA being favoured over LA and ALA [104]. The preference of PUFA for the transfer of FA across the placental membrane is known as biomagnification [100]. Furthermore, a high maternal intake of LA during pregnancy in rats affects the placenta FA composition and transport of FA across the placenta by downregulating the FADS1 and FATP4 mRNA expression [55]. Exposure of Swan 71 trophoblast cells in vitro to increasing concentrations of LA (25–1000 µM, out of which 100, 300, and 500 µM are physiologically relevant concentrations of LA) enhances FATP1 and FATP4 expression, while suppressing FABP3 expression, suggesting that these FABPs play an important role in the transfer of FA from mother to fetus across the placenta, especially when LA is in excess [105]. Increased LA consumption during pregnancy in rats influenced fetal plasma FA composition without affecting placental weight [55]. Total fat content and LA:ALA ratio in maternal diet in a rat model influenced the placental FA composition by decreasing the gene expression of FATP4 [55]. Rats fed with a high LA diet showed decreased ALA in the placenta as compared to those fed on a low LA diet despite the same intake of ALA in both the groups [55,71], suggesting that increased LA in maternal diet during pregnancy influences the uptake of LA and ALA by the placenta. These findings suggest that an elevated maternal LA dietary intake may alter placental metabolism of LA and ALA.

## 12. Correlation between LA and Endocannabinoids

Endocannabinoids (ECs) are important mediators of placentation and play an important role in the development and regulation of the secretion of hormones and maintaining homeostasis in the human body [106,107]. ECs are lipid messengers involved in body weight control and can be formed from the degradation of LA [73]. ECs include the endogenous ligands 2-AG and AEA, which can be generated via LA metabolism and act predominantly via the cannabinoid receptor 1 (CNR1) and cannabinoid receptor 2 (CNR2) [108]. LA may affect physiological functions and processes during fetal development by modulating ECs [109]. Increasing dietary LA to 8% of total energy intake elevated AA levels in the liver and enhanced obesity in pregnant mice compared to a diet of 8% LA supplemented with 1% of ALA. Maternal LA diet of 6.21% of energy alters the CNR2 in maternal and fetal cardiac tissues in males, as compared to a diet of 1.44% of energy in Wistar Kyoto rats [110]. ECs are associated with obesity, inflammation, cardiac functions and lipid and glucose metabolism [111,112]. The link between FA intake and ECs during pregnancy requires further investigation.

## 13. Effect of LA in Maternal Diet during Pregnancy on Offspring Weight during Postnatal Period

In addition to affecting birth weight, offspring of wild-type female mice fed on a high LA diet (LA:ALA—59:1) during pregnancy were 50% heavier at weaning as compared to those fed on isocaloric diet (LA:ALA—2:1) [69]. Nevertheless, when the offspring of wild-type female mice, nourished with a conventional diet (comprising high carbohydrate and low fat content) throughout pregnancy, were subsequently provided with a high-fat diet abundant in LA or an isocaloric diet post-weaning, no notable variance in offspring body weight was observed [69]. Research has also indicated that an increased proportion of LA and ALA in the dietary intake of pregnant women living in Germany is associated with greater fat accumulation in infants at the age of one year [87]. This underscores the significance of maternal diet during gestation. The studies characteristics are summarized in Table 1.

## 14. Correlation between Maternal Dietary LA during Pregnancy and Obesity and Various Metabolic Diseases in Children

A high dietary LA intake during pregnancy has been demonstrated to have a significant influence on the risk of childhood obesity, increasing the risk of various chronic diseases such as hypertension, dyslipidaemia, chronic inflammation, hyperinsulinaemia, endothelial dysfunction, and increased blood clotting tendency in humans [116]. The findings of this study are supported in a rodent model, where feeding female mice during pregnancy with an increased LA and ALA ratio (28:1) in a diet led to a higher body weight in the male offspring, showing the sensitivity of offspring fat deposition (measured by total fat mass and epididymal fat pad weight) to maternal dietary intake of LA [69,113]. Furthermore, an increase in LA (LA 6.21%) in the maternal diet during pregnancy significantly altered the cannabinoid receptors in cardiac tissues of female offspring, potentially leading to deleterious effects on cardiac functions in later life [110].

A study in the Netherlands on 234 mother–child pairs observed that an increase in maternal dietary LA during pregnancy is correlated with an increased risk of obesity in children at 7 years of age [117]. In addition, in Germany, a positive relationship was observed between an increased ratio of LA and ALA in cord blood and childhood obesity at age of 10 [118]. It is vital, therefore, to study the long-term consequences of an increased maternal dietary intake of LA during pregnancy on weight gain in adulthood. The studies characteristics are summarized in Table 2.

In addition to increased risk of weight gain in adulthood, excessive maternal LA intake can influence multiple aspects of child health. Elevated LA levels may contribute to chronic inflammation by increasing the production of pro-inflammatory eicosanoids, such as AA, potentially heightening the risk of autoimmune disorders and inflammatory conditions [65]. Neurologically, a high LA intake could disrupt the balance of fatty acids crucial for brain development, potentially affecting cognitive functions, educational outcomes, and behaviour in children [122]. Cardiovascular health may be impacted, with excessive LA potentially influencing lipid profiles and increasing the risk of future heart disease [123]. Additionally, elevated LA intake has been linked to a higher risk of obesity and metabolic syndrome due to its effects on fat metabolism and insulin sensitivity [124]. Understanding these long-term consequences underscores the importance of LA within maternal diets during pregnancy to optimize health outcomes for mothers and offspring.

## 15. Summary

In both animal and human studies, increased maternal intake of LA during pregnancy has been linked to potential impacts on fetal growth, which could predispose individuals to metabolic disorders later in life. Elevated dietary exposure to LA above recommended levels during pregnancy may elevate inflammatory markers in maternal blood, promoting increased production of AA and the transfer of fatty acids across the placenta. Animal studies indicate that LA’s role in weight gain is tied to its conversion to AA via prostacyclin pathways. Rat studies suggest that diets high in LA and low in ALA lead to weight gain across generations, although these LA levels are much higher than typical human consumption.

LA intake and birth weight response in humans present a mixed picture. Some studies suggest an inverted U-shaped correlation between LA intake and birth weight, with low and high intake levels both associated with low birth weight. Other studies link high maternal LA intake to decreased birth weight and increased rates of small for gestational age babies. Additionally, inconsistencies exist regarding LA’s impact on offspring adiposity measures in different populations. Moreover, investigations into the relationship between LA intake, birth weight, and fetal growth highlight the importance of the LA to ALA ratio during pregnancy. An increased LA to ALA ratio has been linked to decreased placental blood flow, increased blood viscosity, and inadequate fetal growth, potentially leading to metabolic diseases in infancy and adulthood.

An increase in dietary exposure of LA above the recommended nutritional reference values (8 g/day) during pregnancy may increase the inflammatory markers in maternal blood with increased production of AA and transfer of FA across placenta.

## 16. Current Knowledge Gap

Previous animal and human studies suggest that a high LA diet has significant effects on obesity, inflammatory markers, and birth weight, while research in animals also indicates negative impacts on birth weight and a transfer of fatty acids across placenta [72,77,79]. At this time, the mechanism underlying the observed effects of high maternal dietary intake of LA on fetal growth remains elusive, but emerging evidence suggests a potential involvement of inflammatory pathways. However, the specific interplay between maternal inflammatory markers, the transfer of fatty acids across the placenta, and their collective impact on fetal growth is not fully understood. Addressing this knowledge gap is crucial for unravelling the complexities of prenatal nutrition and its consequences on offspring health. Furthermore, elucidating these mechanisms holds significant clinical relevance, as increased birth weight and growth restriction during pregnancy have been linked to an increased risk of metabolic and cardiovascular diseases in adulthood. Thus, comprehensive investigations into the intricate interactions between maternal dietary factors, inflammatory processes, and fetal development are warranted to inform strategies aimed at optimizing maternal and offspring health outcomes.

## Figures and Tables

**Table 1:** Summary of key studies examining the effect of maternal linoleic acid diet during pregnancy on offspring’s weight in animals.

Author Name and Year	Intervention	Duration	Species	Outcome
Korotkova et al., 2002 [70]	Sunflower oil (LA ^1^ diet—7% of energy) Linseed oil (ALA ^2^—7% of energy) Soyabean oil (contained both LA and ALA—9:1)	Last 10 days of gestation and throughout the lactation period	Sprague–Dawley rats	Increase in body weight in the female pups of the dams fed the LA or LA/ALA diet at 1 week and 3 weeks of age, as compared to ALA diet.
Massiera et al., 2003 [69]	Corn oil-supplemented diet rich in linoleic acid (LA diet) A mixture of corn oil and perilla oil rich in α-linolenic acid (LA/ALA diet)	Female mice were fed for 4 weeks before mating, and during pregnancy and lactation period. Pups of mice were fed the same diet after weaning until 22 weeks of age.	Wild type and *ip-r^−/−^* mice (genetically modified mice that lacks IP3R gene)	Body weight of wild-type mice fed on LA diet was higher than that of animals fed on LA/ALA diet, whereas there was no difference in body weight of *ip-r^−/−^* mice fed on LA and LA/ALA diet.
Massiera et al., 2010 [113]	LA: 2.2 g per 100 g, ALA: 0.24–0.26 g/100 g (Chow diet) LA: 7.9 g per 100 g, ALA: 0.24–0.26 g/100 g (LA:ALA, ~28:1) (LA—18% of total energy intake and ALA 0.6% of total energy intake) (High fat diet)	From birth to 10 weeks of age	Mice C57BL6/J	Body weight of male pups of adult male and female mice fed on high LA diet was significantly increased at weaning, which persisted into adulthood.
Alvheim et al., 2012 [76]	LA—8% of energy LA—1% of energy	Female mice pregnant for 2 weeks were fed with LA 8% or LA 1%. Male pups continued with the same diet of their respective mothers for 23 days	Mice (C57BL/6j)	Male pups fed on diet containing LA 8% of energy showed increase in body weight. However, reduction in body weight of these pups was seen when fed on diet containing LA 1% of energy.
Alvheim et al., 2013 [114]	Fish oil—LA:ALA—1: 0.4 Soya bean oil—LA:ALA—8:1	16 weeks	Male mice (C57BL/6j)	Significant increase in body weight in mice fed on soya bean oil compared to mice fed on fish oil from week 9 to week 15.
Sleep et al., 2020 [110]	LA—1.44% of energy (low linoleic acid diet). LA—6.21% of energy (high LA diet)	Female rat was fed on either low or high linoleic acid diet for 10 weeks before mating and 20 days of gestation	Wistar Kyoto rats	No change in body weight in male and female fetuses of rats consuming a high linoleic acid diet
Shrestha et al., [115]	LA—1.44% of energy (low LA diet). LA—6.21% of energy (high linoleic acid diet)	Female rats were fed with either low or high linoleic acid diets for 10 weeks before pregnancy and during gestation/lactation.	Female Wistar Kyoto rats	No significant difference in body weight was observed among low and high linoleic acid groups.

^1^ Linoleic acid; ^2^ Alpha linolenic acid.

**Table 2:** Summary of key studies examining the effect of maternal linoleic acid diet during pregnancy on birth weight in humans.

Author Name and Year	Dietary Assessment Methods	Blood Analysis	Location	Outcome
Van Eijsden et al., 2008 [119]	Food Frequency Questionnaire	Yes	Amsterdam	High maternal dietary LA intake resulted in low birth weight
Mani et al., 2016 [77]	Food Frequency Questionnaire	No	India	Inverted U-shaped (low birth weight at high and low maternal dietary LA intake)
Meher et al., 2016 [79]	Food Frequency Questionnaire	Yes	India	High maternal dietary LA resulted in low birth weight
Grootendorst Van-Mil., 2018 [120]	No dietary assessment method	Yes	The Netherlands	High maternal dietary LA resulted in low birth weight
Lee et al., 2018 [80]	24 h food diary	No	Korea	High maternal dietary LA resulted in low birth weight
Phang et al., 2019 [121]	Food Frequency Questionnaire	Yes	Sydney, Australia	No association between maternal LA dietary intake and birth weight.
